# Real-time imaging reveals that lytic polysaccharide monooxygenase promotes cellulase activity by increasing cellulose accessibility

**DOI:** 10.1186/s13068-018-1023-1

**Published:** 2018-02-15

**Authors:** Bo Song, Bingyao Li, Xiaoyan Wang, Wei Shen, Sungjin Park, Cynthia Collings, Anran Feng, Steve J. Smith, Jonathan D. Walton, Shi-You Ding

**Affiliations:** 1 Department of Plant Biology, Michigan State University, East Lansing, MI 48824 USA; 2 MSU-DOE Plant Research Laboratory, Michigan State University, East Lansing, MI 48824 USA; 3 DOE Great Lakes Bioenergy Research Center, Michigan State University, East Lansing, MI 48824 USA; 4 Nanoscience and Nanoengineering Program, South Dakota School of Mines and Technology, Rapid City, SD 57701 USA

**Keywords:** LPMO, CBH I, AFM, Lignocellulose, Biomass, Cellulose, Biorefinery

## Abstract

**Background:**

The high cost of enzymes is one of the key technical barriers that must be overcome to realize the economical production of biofuels and biomaterials from biomass. Supplementation of enzyme cocktails with lytic polysaccharide monooxygenase (LPMO) can increase the efficiency of these cellulase mixtures for biomass conversion. The previous studies have revealed that LPMOs cleave polysaccharide chains by oxidization of the C1 and/or C4 carbons of the monomeric units. However, how LPMOs enhance enzymatic degradation of lignocellulose is still poorly understood.

**Results:**

In this study, we combined enzymatic assays and real-time imaging using atomic force microscopy (AFM) to study the molecular interactions of an LPMO [*Tr*AA9A, formerly known as *Tr*Cel61A) from *Trichoderma reesei*] and a cellobiohydrolase I (*Tl*Cel7A from *T. longibrachiatum*) with bacterial microcrystalline cellulose (BMCC) as a substrate. Cellulose conversion by *Tl*Cel7A alone was enhanced from 46 to 54% by the addition of *Tr*AA9A. Conversion by a mixture of *Tl*Cel7A, endoglucanase, and β-glucosidase was increased from 79 to 87% using pretreated BMCC with *Tr*AA9A for 72 h. AFM imaging demonstrated that individual *Tr*AA9A molecules exhibited intermittent random movement along, across, and penetrating into the ribbon-like microfibril structure of BMCC, which was concomitant with the release of a small amount of oxidized sugars and the splitting of large cellulose ribbons into fibrils with smaller diameters. The dividing effect of the cellulose microfibril occurred more rapidly when *Tr*AA9A and *Tl*Cel7A were added together compared to *Tr*AA9A alone; *Tl*Cel7A alone caused no separation.

**Conclusions:**

*Tr*AA9A increases the accessible surface area of BMCC by separating large cellulose ribbons, and thereby enhances cellulose hydrolysis yield. By providing the first direct observation of LPMO action on a cellulosic substrate, this study sheds new light on the mechanisms by which LPMO enhances biomass conversion.

**Electronic supplementary material:**

The online version of this article (10.1186/s13068-018-1023-1) contains supplementary material, which is available to authorized users.

## Background

Non-food plant biomass is a sustainable source of fermentable sugars for the production of biofuels and chemicals [1–3]. Cellulose, the main component of lignocellulosic biomass, forms rigid microfibrils composed of well-organized linear β-1,4-glucan chains. Due to its homogeneity and abundance, cellulose is of major interest in producing monomeric sugars for biorefineries [2, 4, 5]. However, the cellulose chains in a microfibril are strongly held together by hydrogen bond networks and van der Waals forces, so that the majority of these chains are not readily accessible to cellulases. In addition, in plant biomass, microfibrils are organized into closely associated bundles and are embedded in a non-cellulosic matrix of lignin and hemicelluloses, which further reduce the efficiency of biomass conversion [2, 6].

The most widespread method of cellulose conversion requires three types of cellulases to act synergistically to convert cellulose to glucose: endo-β-1,4-glucanases (EGs) cleave the internal bonds in the cellulose chain, cellobiohydrolases (CBHs) processively hydrolyze cellulose from the chain ends to produce cellobiose, and β-glucosidases (BGs) hydrolyze cellobiose to glucose [7]. Lytic polysaccharide monooxygenases (LPMOs) are a recently discovered class of enzymes that stimulate biomass hydrolysis and hence improve the efficiency of biomass conversion [8–11]. Unlike cellulases that cleave glycosidic bonds by hydrolysis [12], LPMOs are copper-dependent enzymes that lyse polysaccharide chains by oxidation at either the C1 or the C4 carbon of the glucose unit in the presence of an external electron donor [13–17]. However, the mechanism by which the addition of LPMOs to cellulase mixtures enhances the overall yield of glucose has not been clearly elucidated.

Atomic force microscopy (AFM), especially high-speed AFM, can image with sub-nanometer resolution under aqueous conditions at video rate. AFM has been used to visualize the interactions between enzymes and substrates in real time [18]. AFM has also been used to image the structural changes of cellulose after treatment by LPMO showing disintegrating and fibrillation of cellulose microfibrils [19]. However, how the interaction of LPMO with the cellulose surface causes these changes is still not well understood. In this study, we use high-speed AFM to monitor *in situ* interactions of an LPMO (*Tr*AA9A, formerly known as *Tr*Cel61A) and a CBH I (*Tl*Cel7A), alone and together, with bacterial microcrystalline cellulose (BMCC). Our goal was to visualize in real time the molecular motion of LPMO and its effects on the structure of the cellulose surface, to deepen our understanding of LPMO's role in cellulose hydrolysis.

## Results and discussion

**Fig. 1:**
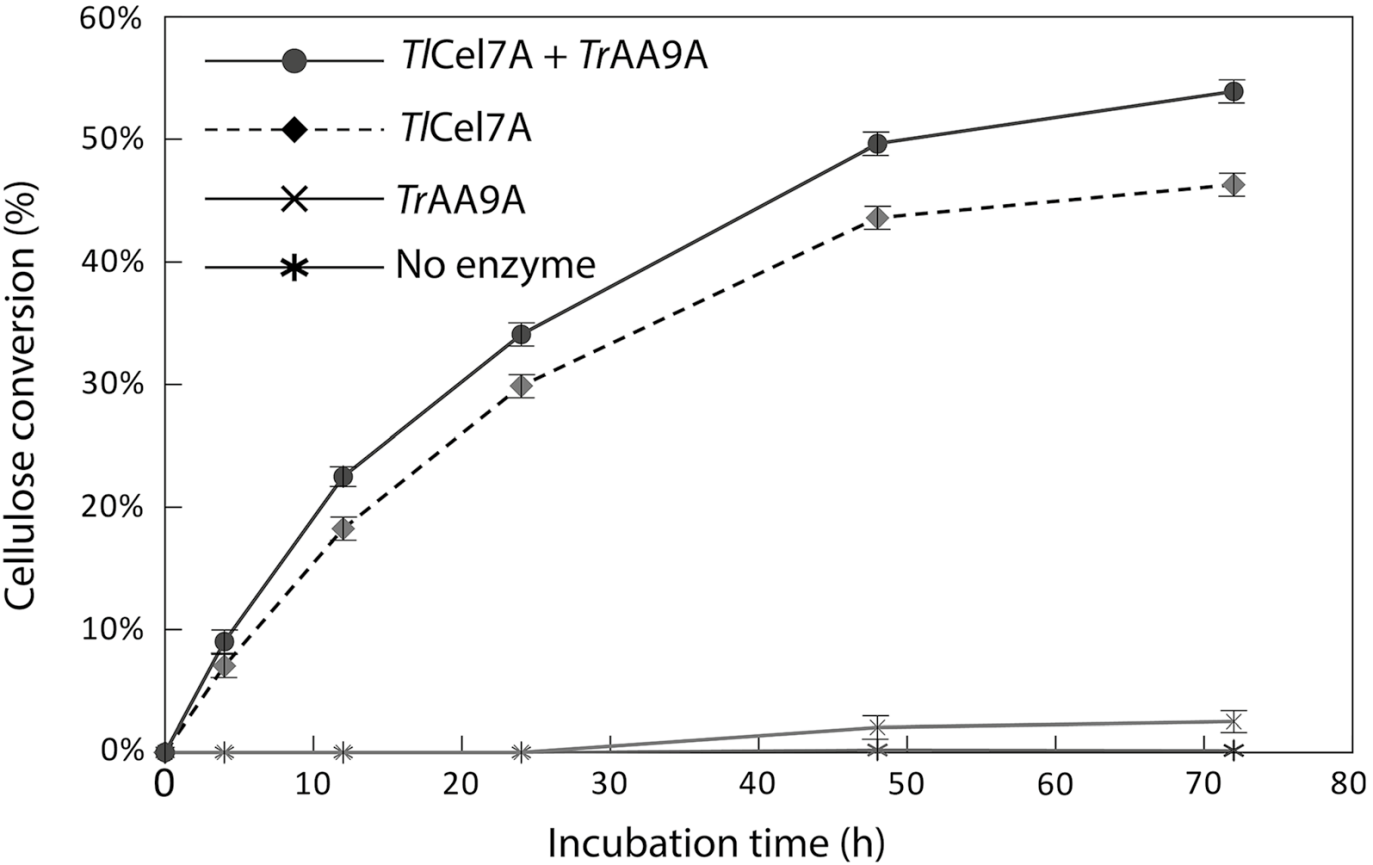
Cellulose conversion by *Tr*AA9A and *Tl*Cel7A. BMCC (2 mg/mL) was treated with *Tl*Cel7A (34 μg/mL) alone or together with *Tr*AA9A (6 μg/mL) in 50-mM sodium acetate buffer (pH 4.8) containing 1-mM l-ascorbic. The reaction was carried out at 50 °C with agitation at 150 rpm. The error bars represent the standard deviation of the triplicates

CBH I is a major cellulase that degrades crystalline cellulose [7, 20]. LPMO has been reported to enhance CBH I hydrolysis of pretreated biomass [8, 11]. Under our experimental conditions, a mixture of *Tr*AA9A and *Tl*Cel7A converted 8% more cellulose (46% compared to 54% of the theoretical maximum yield) than *Tl*Cel7A alone (Fig. 1). This result is consistent with the previous work showing that hydrolysis of cellulose in pretreated corn stover increased approximately 6% by adding a *Thielavia terrestris* LPMO to *T. reesei* cellulase mixtures [8]. Therefore, we assume that the *Tr*AA9A used in this study exhibits similar overall activity in enhancing cellulose hydrolysis by *Tl*Cel7A.

LPMO has been shown to break cellulose chains by oxidation at the C1 and/or C4 position(s) [4, 21, 22]. To further confirm the specific activity of *Tr*AA9A, we used mass spectrometry (MS) to analyze the soluble products after 72 h incubation of *Tr*AA9A with BMCC, and detected small amounts of oxidized forms of cellobiose and cellotriose (Additional file 1: Figure S1), consistent with the previous reports [23].

**Fig. 2:**
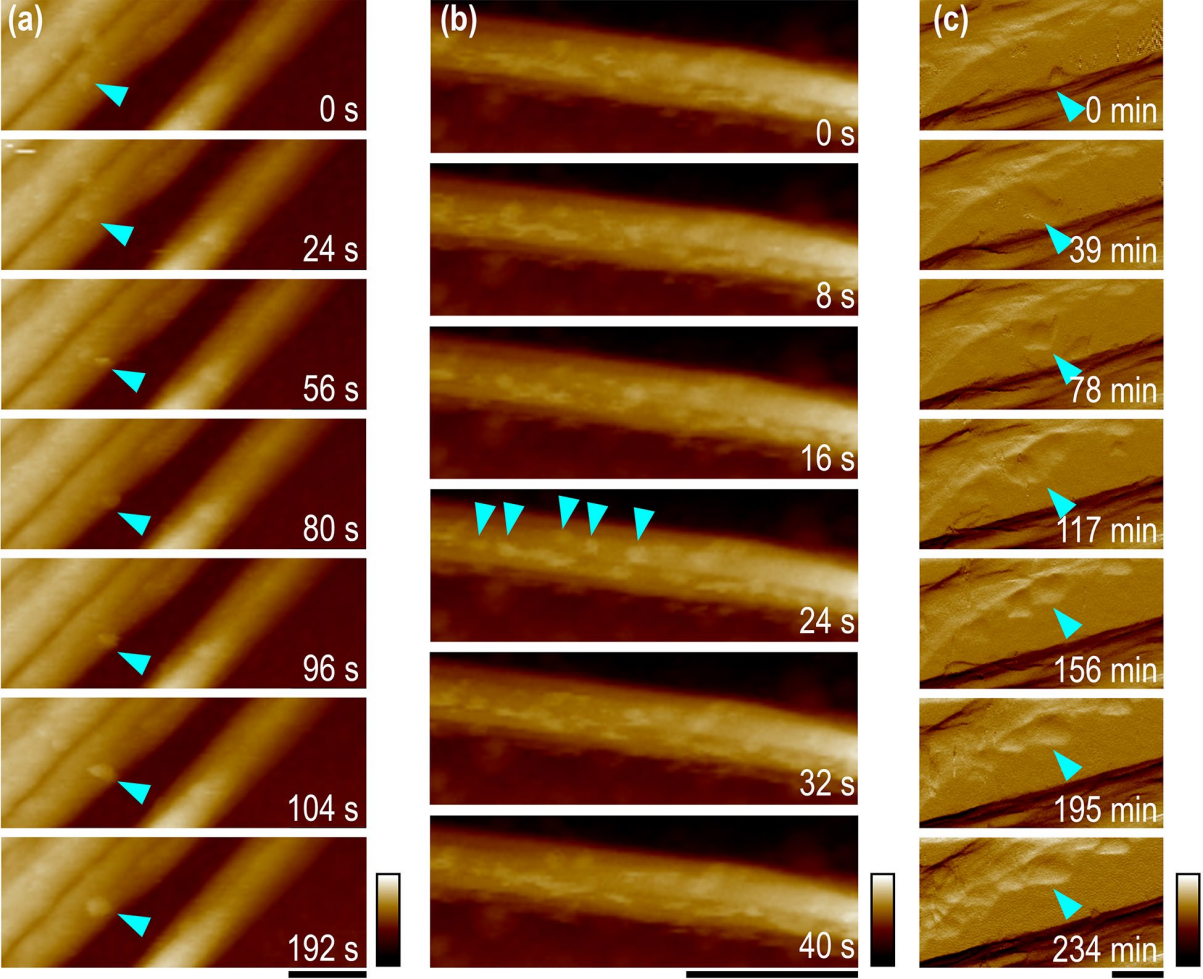
*Tr*AA9A molecules move and diffuse randomly on BMCC. Time-lapse images from Additional file 2: Video S1, Additional file 3: Video S2, Additional file 4: Video S3 show *Tr*AA9A molecules moving across **a**, along **b**, and diffusing **c** between cellulose ribbons. Cyan arrows indicate individual enzyme molecules. Scale bar = 150 nm, and color bars are 55 nm in **a** and **b**, and 100 pN in **c**

To visualize *Tr*AA9A interaction with BMCC under AFM, a small piece of BMCC was transferred onto a glass slide pre-coated with poly-lysine. As the negative control, BMCC was imaged under the same buffer used for the enzyme reactions. We observed primarily large ribbon-like cellulose structures of 30–150-nm width, with a relatively smooth surface. About 10 min after adding *Tr*AA9A, we observed particles of 6–10 nm in diameter accumulating on the surface of the BMCC ribbon (Fig. 2). *Tr*AA9A was predicted to be ~6 nm in diameter based on the structure of *Ta*GH61 [14, 24], and we, therefore, considered these particles to be bound *Tr*AA9A molecules.

Continuous imaging revealed that *Tr*AA9A molecules initially bound randomly to the BMCC surface, and individual *Tr*AA9A molecules exhibited a “stop-and-go” behavior. Interestingly, the enzyme molecules stayed in approximately the same position for a much longer time than they spent moving on the cellulose. *Tr*AA9A moved at 0.25 ± 0.13 nm/s (*n* = 40) in three patterns: in both directions along one cellulose ribbon, across one ribbon, or moving from one ribbon to another (Fig. 2a–c, respectively; details in Additional file 2: Video S1, Additional file 3: Video S2, Additional file 4: Video S3). The observed speed of *Tr*AA9A was one order of magnitude slower than previously observed for CBHI moving on Valonia microcrystalline cellulose (*Tr*Cel7A) [25]. This might be related to the slow rate of oxidization catalyzed by *Tr*AA9A [16]. Interestingly, when the imaging period was extended to ~7 h, some *Tr*AA9A molecules seemed to form into groups that moved together towards a particular cellulose ribbon where eventually many molecules accumulated (Fig. 2c; Additional file 4: Video S3).

Previously, AFM imaging revealed that *Tr*Cel7A binds and reacts on the hydrophobic face of crystalline cellulose [26] and moves processively from the reducing end of the glucan chain to the non-reducing end [25]. In contrast, *Tr*AA9A moves in both directions along a ribbon and also across ribbons. The different binding and movement patterns of *Tr*AA9 and *Tl*Cel7A observed in this study can be explained by the differences in their molecular structures. The binding face of LPMO is flat [14, 24], whereas CBHs like *Tl*Cel7A adopt a tunnel-shape active site that can hold several glucose residues of the cellulose chain and thereby constrain movement in a linear fashion [21, 22, 27, 28].

**Fig. 3:**
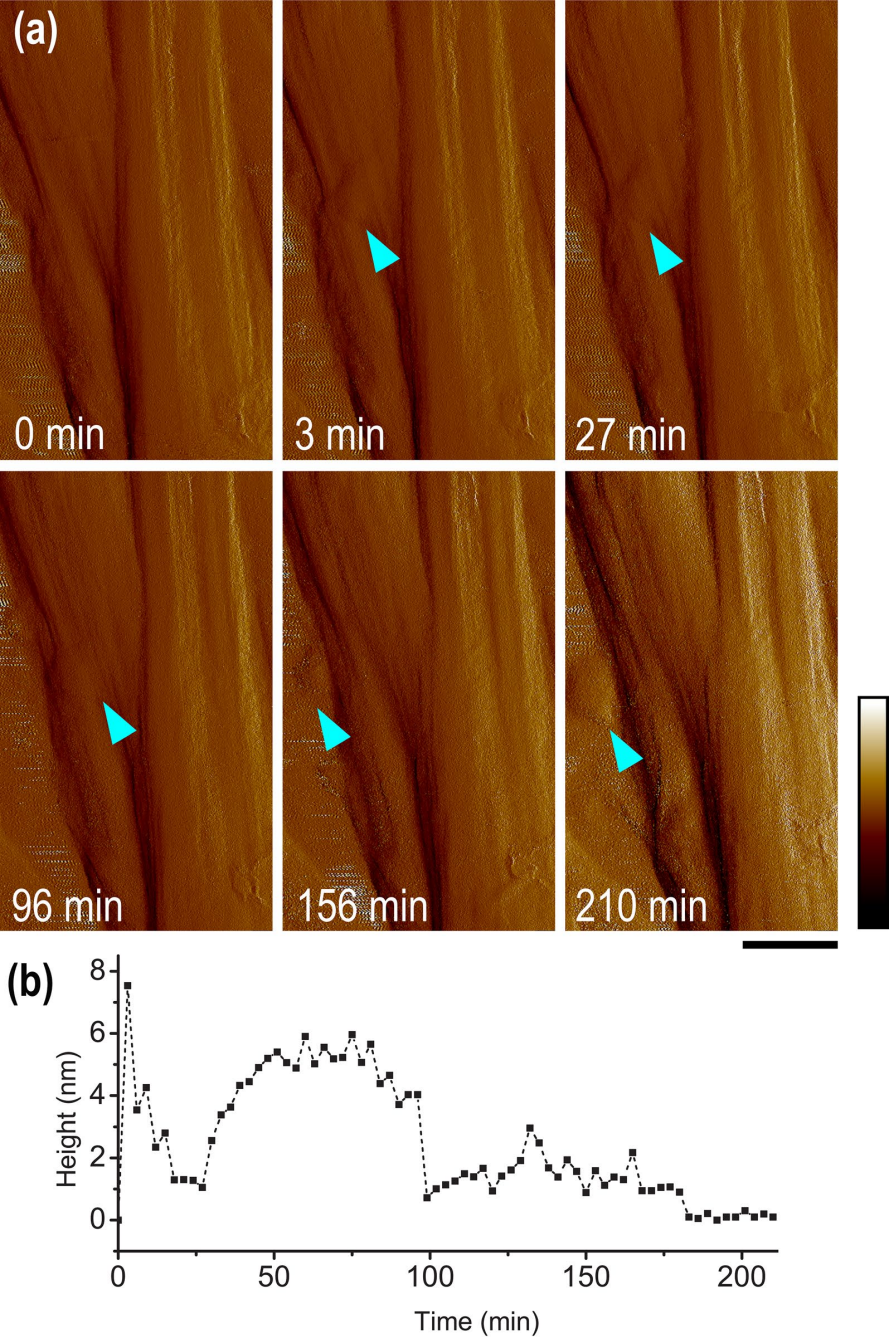
*Tr*AA9A penetrating and moving inside a BMCC ribbon. **a** Time-lapse AFM peak force error images showing *Tr*AA9A (indicated by the cyan arrows) moving in and out of the surface of a cellulose ribbon (See Additional file 5: Figure S2 and Additional file 6: Video S4 for more information). **b** Relative height measured across a *Tr*AA9A molecule during 210 min incubation. Scale bar is 100 nm and color bar is 1.1 nN

By monitoring the height change in the region where individual *Tr*AA9A enzymes crossed, we observed the unexpected phenomenon of enzyme molecules penetrating inside the ribbon (Fig. 3, Additional file 5: Figure S2, and Additional file 6: Video S4). After the addition of *Tr*AA9A, there was an initial increase in height due to the bound enzyme. Subsequently, *Tr*AA9A molecules penetrated inside the cellulose ribbon and were hardly visible after 27 min. The enzyme gradually came back to the surface after 96 min, moved along the cellulose surface, and diffused away after 210 min. As *Tr*AA9A moved, groove-shape features appeared on the cellulose microfibril surface and the existing grooves deepened and widened. As a result, more obvious edges appeared on the cellulose surface after 2-h incubation with *Tr*AA9A (Additional file 7: Figure S3, and Additional file 8: Video S5).

**Fig. 4:**
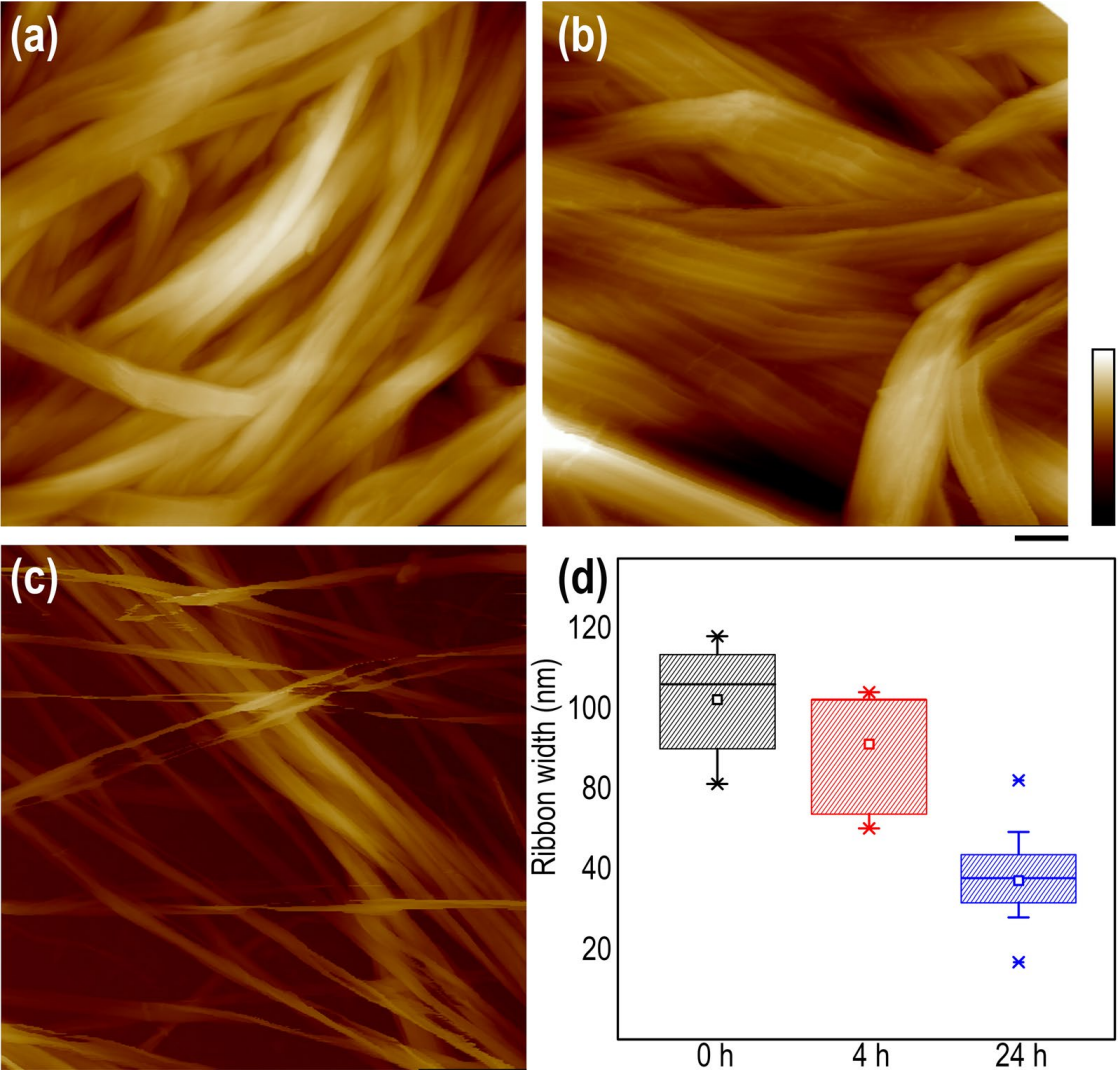
Disassembly of BMCC ribbon into smaller fibrils by *Tr*AA9A. Atomic force micrographs of BMCC before **a**, after 4-h **b** and after 24-h **c** incubation with *Tr*AA9A. **d** Width (and standard deviation) of BMCC microfibrils before and after *Tr*AA9A treatment. Scale bar is 150 nm and color bar is 200 nm

**Fig. 5:**
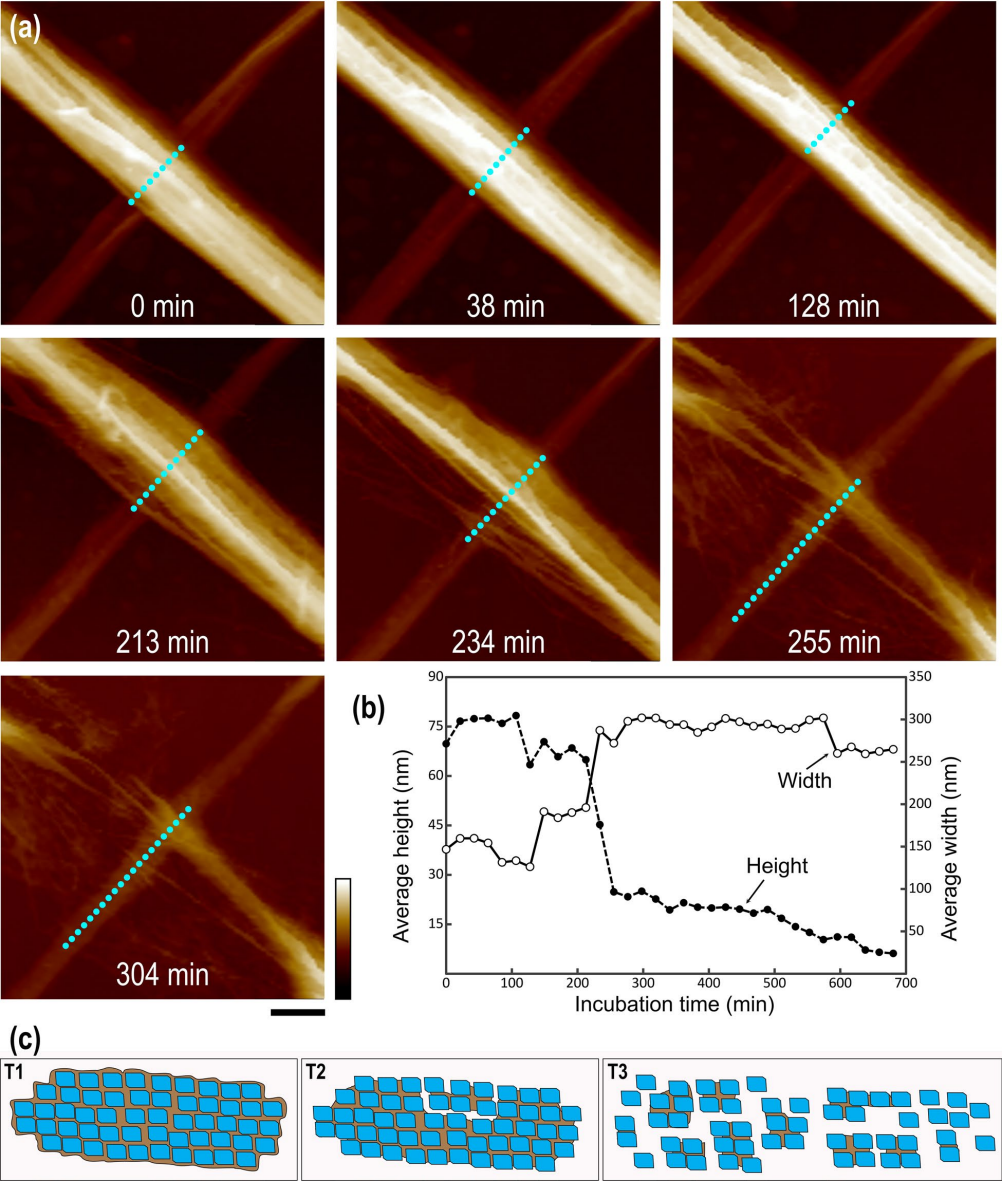
Synergism between *Tr*AA9A and *Tl*Cel7A during hydrolysis of BMCC. **a** Time-lapse images from Additional file 10: Video S6 showing changes in the appearance of the cellulose ribbon during incubation with *Tr*AA9A and *Tl*Cel7A. **b** Height and width measurements. Cyan dash lines in **a** indicate the overall width change of a BMCC ribbon during enzymatic treatment. Scale bar is 50 nm, and color bar is 100 nm

The width of cellulose microfibrils was measured during incubation with *Tr*AA9A (Fig. 4). After 4-h treatment, the average width of the cellulose ribbons slightly decreased from 101.8 ± 14.9 nm (*n* = 20) to 89.8 ± 16.7 nm (*n* = 16), but the surface roughness increased greatly, with obvious grooves and edges observed on the surface (Fig. 4b) compared to a relatively smooth surface before treatment (Fig. 4a). After 24-h incubation, we observed splitting of the ribbon-like cellulose microfibrils along its long axis into smaller microfibrils of 52.9 ± 11.7 nm (*n* = 24) in width, which was approximately 52% of their original ribbon width. Similar structural change was also observed recently [19] using bleached softwood Kraft pulp as the substrate treated with LPMO. We postulate that the ability of *Tr*AA9A to penetrate inside the cellulose ribbon leads to the dividing of a large cellulose ribbon into multiple smaller microfibrils. Considering that a BMCC ribbon is composed of multiple cellulose elementary fibrils (CEF) that can be as small as 2–4 nm [29, 30], it is possible that *Tr*AA9A oxidation disrupts the glucan chains between these CEFs. We further studied the overall BMCC structure changes by incubating *Tl*Cel7A with and without *Tr*AA9A. After 72-h incubation with both enzymes, BMCC was broken into small pieces. In contrast, treatment with *Tl*Cel7A alone resulted in softening of BMCC floating on the top of the reaction buffer. There was no significant structural change of BMCC viewed by eye after *Tr*AA9A treatment alone (Additional file 9: Figure S4). AFM was used to further visualize the structural changes of individual BMCC ribbon after treatment of these two enzymes at the nanometer scale (Fig. 5). For the first 20 min, the average height and width of the cellulose ribbon slightly increased, which was likely due to the binding of *Tr*AA9A and *Tl*Cel7A onto the cellulose surface. After 7-h incubation, the height of the BMCC ribbon decreased, while the width increased. A significant decrease in height and increase in width was observed from 106 to 149 min, which was concomitant with the disintegration of the large cellulose ribbon into small microfibrils. Between 213 and 255 min, the cellulose ribbon completely separated into small microfibrils of ~4 nm in diameter, as the ribbon height continued to decrease and the width to increase (Fig. 5a, details in Additional file 10: Video S6). The diameter of the small microfibril (~4 nm) indicated that they could contain an individual or a bundle of at most a few fundamental CEFs [29, 30].

After 4 h of incubation, the resulting cellulose microfibrils were significantly thinner when treated by *Tr*AA9A together with *Tl*Cel7A (Fig. 5a) than when treated by *Tr*AA9A alone (Fig. 4). Interestingly, cellulose hydrolysis by the cellulase mixture composed of *Tl*Cel7A, EG, and BG increased from 79 to 87% when using *Tr*AA9A-pretreated BMCC, compared with untreated BMCC (Additional file 11: Figure S5). This enhancement is similar to the results for *Tr*AA9A and *Tl*Cel7A added together to untreated BMCC, suggesting that the synergy of *Tr*AA9A and cellulases during cellulose hydrolysis may not be attributed to the direct molecular interaction between these enzymes.

## Conclusions

We used high-speed AFM imaging to visualize in real-time the enzymatic hydrolysis of cellulose by cellulases and an LPMO, *Tr*AA9A, in this study. Our results are consistent with the previous studies, but further show that cellulases, i.e., CBHs and EGs, are responsible for the majority of cellulose hydrolysis, and that LPMOs promote their efficiency. We observe that LPMO reacts specifically to disintegrate large cellulose ribbons into small microfibrils with little oxidized sugar products, whereas CBHI has been considered to be a “peel-off” process [26, 31]; we hypothesize that LPMO and CBHI may attack different structures of cellulose. BMCC ribbons are considered to contain amorphous cellulose and CEFs that are composed of well-organized cellulose glucan chains [29, 30]. It is likely that LPMO attacks only the amorphous proportion of cellulose and increases the surface accessibility of CEFs for cellulase enzymes. If this is true, LPMO would not enhance cellulase activity when cellulose accessibility is already at a maximum. Indeed, it has been previously demonstrated that the degree of LPMO enhancement is negatively correlated with cellulose accessibility [9]. Similarly, LPMO has been found to increase the degradation of insoluble xylan [32], one of the major matrix polysaccharides in the plant cell wall, suggesting that LMPO can oxidize sugars in different amorphous polysaccharides, thus exposing the well-organized cellulose microfibrils to be hydrolyzed by cellulases.

## Methods

### General chemicals

All chemicals and reagents, unless specifically noted, were purchased from Sigma-Aldrich (St. Louis, MO).

### Bacterial strain and medium

*Gluconacetobacter xylinus* (also known as *Komagataeibacter xylinus*) strain ATCC 53524 from the American-Type Culture Collection (Manassas, VA) was maintained on Hestrin and Schramm (HS) medium containing 1.5% agar. HS medium contains 20-g/L glucose, 5-g/L peptone, 5-g/L yeast extract, 2.7-g/L Na_2_HPO_4_, and 1.15-g/L citric acid monohydrate. The pH was adjusted to 6.0 with NaOH or HCl.

### Enzymes

*Tl*Cel7A from *Trichoderma longibrachiatum* (Catalog No. E-CBHI) was purchased from Megazyme, Ltd., Bray, Ireland. *Tr*AA9A (GenBank CAA71999), endo-β-1,4-glucanase (EG; AAA34212) and β-glucosidase (BG; AAA18473) from *T. reesei* were expressed in *Pichia pastoris* as previously described [33]. Pronase E (Catalog No. P2714) was purchased from Sigma-Aldrich (St. Louis, MO).

### Production of BMCC

The bacterial microcrystalline cellulose (BMCC) was produced based on the previous study with modifications [34, 35]. HS medium (50 mL) in a 250-mL flask was inoculated with *G. xylinus* colonies and incubated at 30°C for 1–3 days in static until cellulose pellicle was visible. The culture was vigorously shaken for 30 min to release active cells embedded in the pellicle. Then, 450-mL fresh HS medium in a plastic tray (18 × 6 × 2.5 in.) was inoculated with the culture broth and incubated in stationary for another 3–5 days until the cellulose pellicle was grown on the entire medium surface.

The cellulose pellicle was collected by filtering through 2-layer Miracloth. Then, the pellicle was washed repeatedly with distilled deionized water (ddH_2_O) to rinse off the medium as much as possible. Washed cellulose was boiled in 1% NaOH with stirring for 30 min to remove remaining bacteria. Finally, alkali-treated cellulose was washed several times with ddH_2_O until the water pH reached 7.0, freeze-dried, and cut to 1-cm strip for enzymatic degradation. Native BMCC without alkali treatment was stored in 0.02% sodium azide at 4 °C for AFM imaging.

### Preparation of BMCC samples

The BMCC sample was prepared by a hand-cutting of fresh and never dried BMCC film. Only thin layers with approximated 10–50-μm thickness were used in the experiment. A bright field microscopy was used to select a thinner sample with relative uniform surface. After washing by deionized water several times and placed on a poly-lysine-coated glass slide (Thermo Fisher Scientific Inc, Waltham, MA, USA) with deionized water. The samples were kept in the water for all of the imaging and measurement process.

### AFM operation

All the AFM experiments were conducted at room temperature on a Dimension AFM with Nanoscope controller V (Fastscan, Bruker Nano, Santa Barbara, CA. USA) with an acoustic and vibration isolation system. Probes we used were SCANASYST-FLUID+ (Bruker, Camarillo, CA USA) for imaging under fluid. The AFM operation software (Nanoscope V9.1) was used to control the scan size, setpoint, and gain. Before AFM imaging, the scanner has been carefully calibrated using calibration kit (Bruker, Camarillo, CA, USA) to make sure that all the measurements are very close to their actual value. To dynamically capture the movement of enzyme molecules, the scan rate was normally set as 10–20 Hz for the continuous observation with low resolution (256 × 128 pixels or 128 × 64 pixels). However, when we conducted the imaging on single *Tr*AA9A penetration experiment, we lowered the scan rate to 2 Hz to obtain images with better resolution. For static observations, the scan rate was 1 Hz with the resolution of 1024 × 1024 pixels.

### AFM measurement and image processing

All off-line data analysis was based on the Nanoscope Analysis v1.8 software (Bruker Nano, Santa Barbara, CA. USA). The height and peak force error images were analyzed using plane fit filter at one order for images presented in all figures. The color bar was manually modified according to the best presence of each image. The images used for height and width measurement were raw data without any processing and all width and height measurements were conducted with the “section” function in the Nanoscope Analysis software.

### Preparation of TrAA9A-treated BMCC

2-mg/mL BMCC was incubated with 100-μg/mL *Tr*AA9A, 1-mM _L_-ascorbic acid, and 0.02% sodium azide for 72 h in 50-mM pH 4.8 sodium acetate buffer. All reactions in triplicate were conducted at 150 rpm, 50 °C in shaking incubator. After 72-h incubation, BMCC residue was recovered by filtering through two layers of Miracloth (Calbiochem, San Diego, CA). Filtrates from all reactions were saved for *Tr*AA9A product analysis. Then, *Tr*AA9A remained on cellulose residue was removed according to the previous study with modifications [36]. The residue was first washed several times with ddH_2_O. Then, it was incubated with 10-mg/mL Pronase E in 50-mM pH 7.5 Tris buffer overnight at 37 °C, 100 rpm for complete proteolysis of remaining *Tr*AA9A. Next, BMCC residue was collected by filtering through two layers of Miracloth, washed with ddH_2_O, 1 M NaCl, and ddH_2_O again to remove Pronase E. Finally, the BMCC residue was freeze-dried and stored at 4 °C.

### TrAA9A product analysis by LC–MS

Filtrate obtained after 72-h reaction with *Tr*AA9A was centrifuged in 70% ethanol for 20 min at 4 °C to precipitate *Tr*AA9A and supernatant was collected. Filtrate of BMCC incubated under the same condition without *Tr*AA9A was also prepared in the same way for mass spectrometry analysis. Samples were analyzed on a Waters Xevo G2-XS Q-TOF system coupled to a Waters I-Class UPLC system. Carbohydrates were separated by an ACQUITY UPLC BEH Amide column (2.1 × 100 mm, 1.7 μm) maintained at 40 °C, with the injection volume at 10 μL. Solvent A was 10-mM ammonium formate, and solvent B was 100% acetonitrile. The solutes were eluted at 0.2 mL/min starting at 95% B, followed by a linear gradient to 35% B over 14 min. The proportion of solvent B was maintained at 35% for 2 min and then increased back to 95% and kept for 4 min for re-equilibration.

The operation condition for mass spectrometer was capillary voltage of 3.0 kV, sample cone voltage of 80 V, source temperature of 100 °C, desolvation temperature of 350 °C, and desolvation gas flow of 600 L/h. Mass spectra were acquired in positive ion mode across the 50–2000-m/z range. Data processing was performed using the MassLynx software (version 4.1, Waters).

### Enzymatic degradation of BMCC

To test the synergism between *Tr*AA9A and *Tl*Cel7A on cellulose degradation, 2-mg/mL BMCC was incubated with 34-μg/mL *Tl*Cel7A and 6-μg/mL *Tr*AA9A, alone or together, in 10-mL reactions containing 1-mM _L_-ascorbic acid and 0.02% sodium azide. Reactions were conducted in triplicate in 50-mM sodium acetate, pH 4.8, for 72 h at 150 rpm, 50 °C. To detect cellulose conversion rate, 100-μL supernatant was incubated with 40-μg/mL β-glucosidase in 50-mM pH 4.8 sodium acetate buffer for 30 min at 50 °C. Free glucose was measured by enzyme-linked colorimetry as described [33].

To test the digestibility of *Tr*AA9A-treated cellulose, *Tr*AA9A-treated BMCC was compared against untreated BMCC for enzymatic hydrolysis at pH 4.8. For all reactions, cellulose loading was 2 mg/mL and cellulase mixture loading 100 μg/mL, which contained *Tl*Cel7A, EG, and BG with 6:3:1 ratio. Each reaction in triplicate was carried at 150 rpm, 50 °C in 0.02% sodium azide. Glucose yield was measured during 72-h incubation.

## Additional files

**Additional file 1: Figure S1.** ESI-MS analysis of oxidized cellobiose and cellotriose released from BMCC by *Tr*AA9A. BMCC (2 mg/mL) was incubated with 100 μg/mL *Tr*AA9A and 1 mM L-ascorbic acid in 50 mM sodium acetate, pH 4.8, for 72 h at 50 °C and 100 rpm. The reactions were conducted in triplicate. As the negative control, BMCC alone was incubated for 72 h under the same condition. (a) Small amount of cellobiose was detected in the negative control. (b), (c), and (d) Cellobiose (DP2), cellotriose (DP3), and two oxidation products derived from DP2 and DP3 were detected in all replicates of *Tr*AA9A-containing samples. Lytic polysaccharide monooxygenase (LPMO) degrades cellulose to cello-oligosaccharides and oxidized cello-oligomers. Three types of LPMOs were described based on their oxidation products. Type 1 enzymes oxidize the C1 carbon of the glucose unit and produce aldonolactone and its hydrated product aldonic acid [15, 16, 37, 38]. Type 2 enzymes oxidize C4 carbon and generate gem-diol intermediate and 4-ketoaldose [10, 23, 39]. Type 3 oxidizes both C1 and C4 carbons and could generate C1 oxidation product, C4 oxidation product, and C1–C4 double-oxidized products [37, 39, 40–43]. C1 or C4 oxidation alone can lead to the breakage of cellulose chain. Products of *Tr*AA9A were analyzed by mass spectrometry. After 72 h incubation, BMCC alone produced a small amount of cellobiose (DP2) but no other cellodextrin or oxidized sugar (Fig. S1a). In contrast, *Tr*AA9A generated cellobiose (DP2), cellotriose (DP3), and two types of oxidation products derived from DP2 and DP3 after 72 h reaction (Fig. S1b, c, d). Cellodextrin and oxidized sugar products were all associated with Na^+^ in mass spectrum probably because the reactions were conducted in sodium acetate buffer [44]. Although there was a small amount of cellobiose in the negative control, its relative intensity is much lower than that from *Tr*AA9A-containing reactions. Therefore, cellobiose is probably one of the products of *Tr*AA9A. In contrast to many studies, cello-oligomer and oxidized cello-oligomer of higher degree of polymerization (DP) were not detected after 72 h *Tr*AA9A reaction. One possible explanation is that *Tr*AA9A might also be active on cello-oligosaccharide [23] and that cellodextrins of higher DP were degraded after 72 h reaction. Due to the identical masses between gem-diol and aldonic acid (DPx + 16 amu, DPx represents cellodextrin), and between ketoaldose and aldonolactone (DPx – 2 amu), MS analysis alone cannot determine the type of *Tr*AA9A activity [23].

**Additional file 2: Video S1.** AFM (Height) of *Tr*AA9A molecules moved and diffused across on BMCC cellulose ribbons for approximately 10 min. The images were recorded at 128 x 64 pixels, and 17-Hz scan rate. The height scale was fixed at 100 nm. The movie is played back at ~650 times of actual speed.

**Additional file 3: Video S2.** AFM (Height) of *Tr*AA9A molecules moved and diffused along on BMCC cellulose ribbons for approximately 10 min. The images were recorded at 128 x 42 pixels, and 17-Hz scan rate. The height scale was fixed at 35.5 nm. The movie is played back at ~650 times of actual speed.

**Additional file 4: Video S3.** AFM (Peak force error) of *Tr*AA9A molecules moved and jumped between BMCC cellulose ribbons for approximately 6 hours. The images were recorded at 256 x 256 pixels, and 1.5-Hz scan rate. The height scale was fixed at 1.3 nN. The movie is played back at ~1800 times of actual speed.

**Additional file 5: Figure S2.** *Tr*AA9A penetrating and moving inside BMCC ribbon. Time-lapse images showing *Tr*AA9A (indicated by the cyan arrows) moving in and out of the surface of a cellulose ribbon during 210 min incubation. Scale bar is 100 nm and color bar 75 nm.

**Additional file 6: Video S4.** AFM (Peak force error) of *Tr*AA9A molecules penetrated and moved inside BMCC cellulose ribbons for approximately 3 hours. The images were recorded at 256 x 256 pixels, and 1.5-Hz scan rate. The height scale was fixed at 0.8 nN. The movie is played back at ~1800 times of actual speed.

**Additional file 7: Figure S3.** Changes in morphology of BMCC ribbon when incubated with *Tr*AA9A (indicated by cyan arrows) during 2 h continuous AFM observation. Pictures were taken from Video S5. Scale bar is 50 nm. Color bar is 50 nm and 830 pN in height (left) and peak force error (right) channels, respectively.

**Additional file 8: Video S5.** AFM (Height) of *Tr*AA9A molecules moved and caused morphology change of BMCC cellulose ribbon for approximately 2 h. The images were recorded at 128 x 64 pixels, and 17-Hz scan rate. The height scale was fixed at 50 nm. The movie is played back at ~650 times of actual speed.

**Additional file 9: Figure S4.** Morphology change of BMCC after 72 h incubation with or without enzymes. Reaction conditions were the same as Fig. 1. BMCC was cut to 1-cm strip for enzymatic digestion. Cellulose loading was 2 mg/mL, *Tl*Cel7A 34 μg/mL, and *Tr*AA9A 6 μg/mL. Reactions were conducted in triplicates in 50 mM, pH 4.8, sodium acetate buffer at 150 rpm and 50°C. No obvious morphological change of cellulose strip after *Tr*AA9A treatment was observed. In contrast, after 72 h incubation with *Tl*Cel7A, the length of the strip shortened to approximately half of its original length with almost 50% cellulose degradation (Fig. 1). Surprisingly, after 72 h incubation with both *Tr*AA9A and *Tl*Cel7A, the cellulose gel was broken into small insoluble particles (mostly floating on surface in the picture).

**Additional file 10: Video S6.** AFM (Height) of morphological changes of cellulose ribbon during incubation with *Tr*AA9A and *Tl*Cel7A for approximately 6 hours. The images were recorded at 256 x 256 pixels, and 10-Hz scan rate. The height scale was fixed at 100 nm. The movie is played back at ~1000 times of actual speed.

**Additional file 11: Figure S5.** Comparison of cellulase hydrolysis of untreated and *Tr*AA9A-treated BMCC. Cellulose loading was 2 mg/mL and total cellulase loading was 100 μg/mL with *Tl*Cel7A: EG: BG ratio of 6:3:1. The reactions in triplicate were conducted in 50 mM, pH 4.8, sodium acetate buffer at 150 rpm, 50°C for 72 h.

## Abbreviations

LPMO: lytic polysaccharide monooxygenase
CBH: cellobiohydrolase
*Tr*AA9A: a lytic polysaccharide monooxygenase from *Trichoderma reesei*
*Tl*Cel7A: a cellobiohydrolase I from *Trichoderma longibrachiatum*
BMCC: bacterial microcrystalline cellulose
EG: endo-β-1,4-glucanase
BG: β-glucosidase
