# Supplementary figures and images for: Real-time imaging reveals that lytic polysaccharide monooxygenase promotes cellulase activity by increasing cellulose accessibility
Source: Biotechnol Biofuels. 2018 Feb 15;11:41. doi: 10.1186/s13068-018-1023-1 (PMC5815216; doi:10.1186/s13068-018-1023-1)

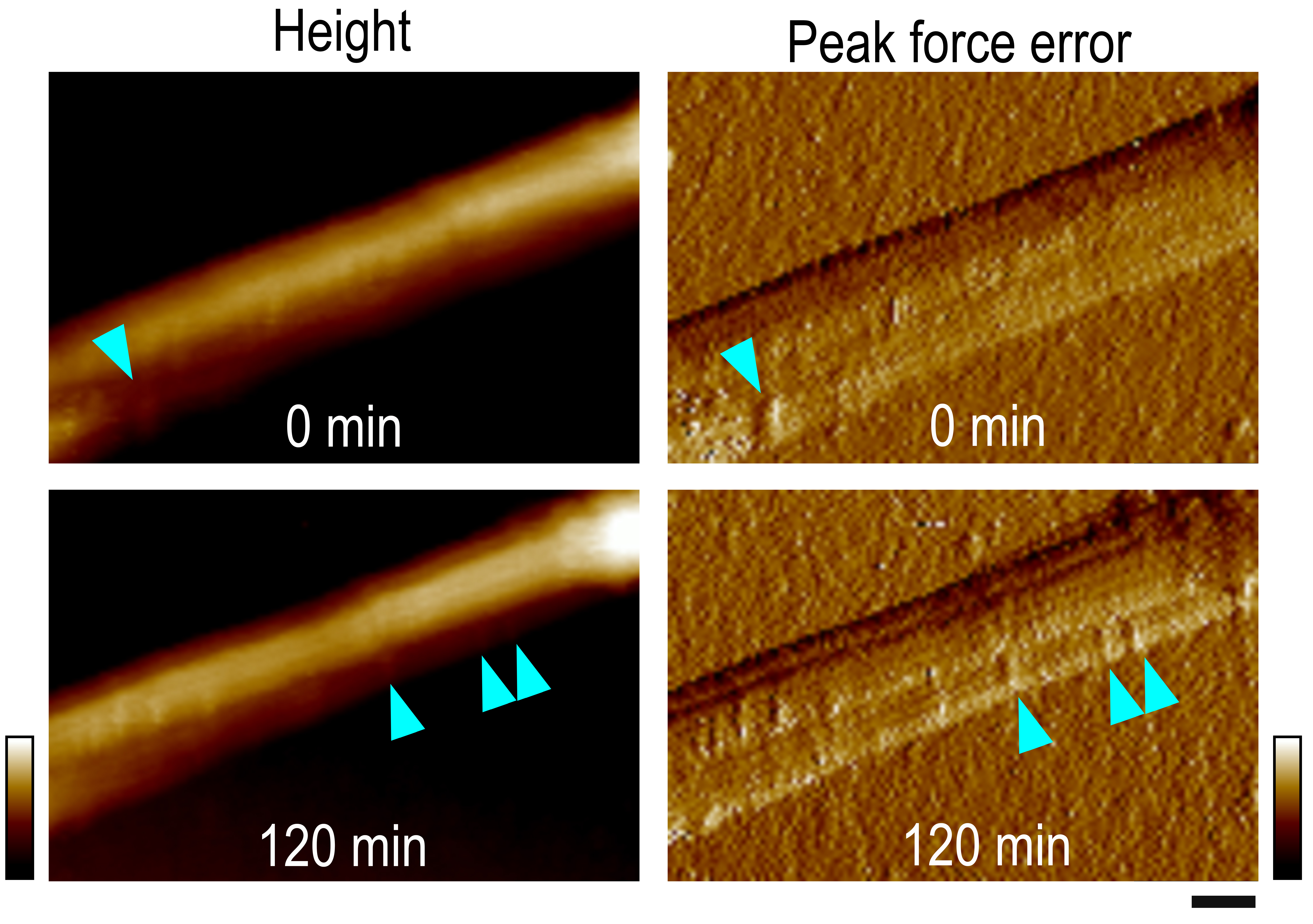

Supplement: Supplementary file 7 — Additional file 7: Figure S3. Changes in morphology of BMCC ribbon when incubated with TrAA9A (indicated by cyan arrows) during 2 h continuous AFM observation. Pictures were taken from Video S5. Scale bar is 50 nm. Color bar is 50 nm and 830 pN in height (left) and peak force error (right) channels, respectively. [file 13068_2018_1023_MOESM7_ESM.jpg]

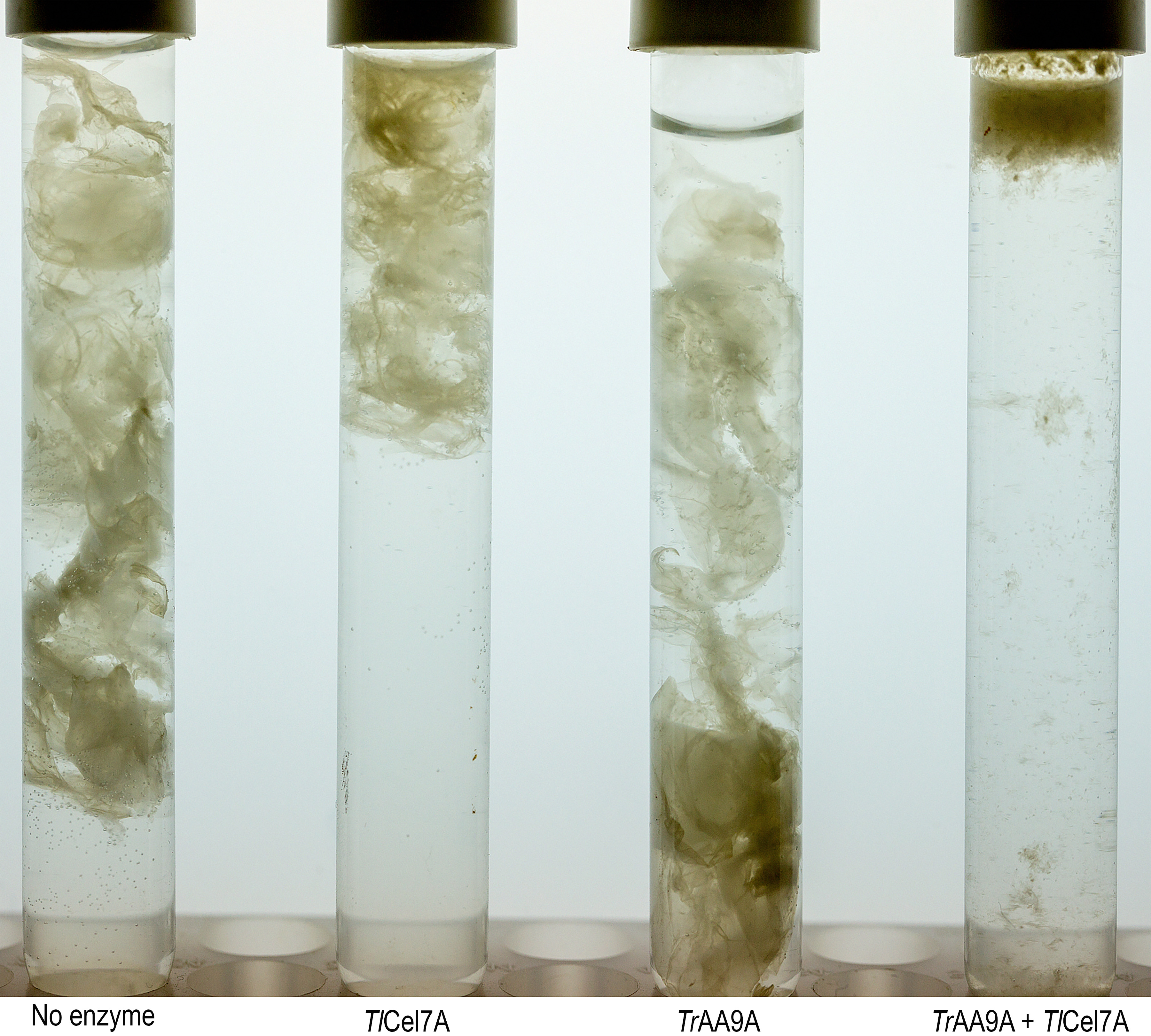

Supplement: Supplementary file 9 — Additional file 9: Figure S4. Morphology change of BMCC after 72 h incubation with or without enzymes. Reaction conditions were the same as Fig. 1. BMCC was cut to 1-cm strip for enzymatic digestion. Cellulose loading was 2 mg/mL, TlCel7A 34 μg/mL, and TrAA9A 6 μg/mL. Reactions were conducted in triplicates in 50 mM, pH 4.8, sodium acetate buffer at 150 rpm and 50°C. No obvious morphological change of cellulose strip after TrAA9A treatment was observed. In contrast, after 72 h incubation with TlCel7A, the length of the strip shortened to approximately half of its original length with almost 50% cellulose degradation (Fig. 1). Surprisingly, after 72 h incubation with both TrAA9A and TlCel7A, the cellulose gel was broken into small insoluble particles (mostly floating on surface in the picture). [file 13068_2018_1023_MOESM9_ESM.jpg]

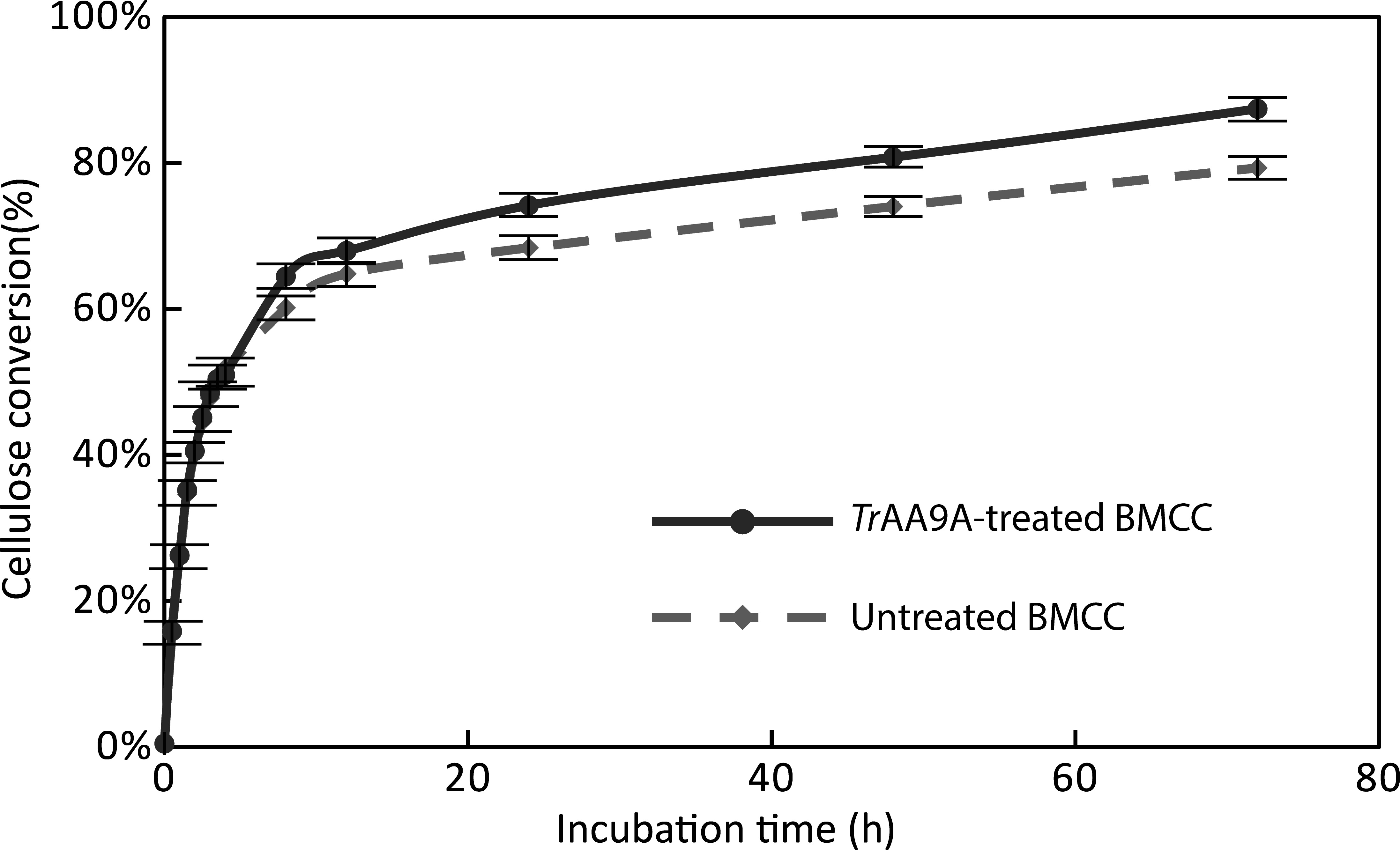

Supplement: Supplementary file 11 — Additional file 11: Figure S5. Comparison of cellulase hydrolysis of untreated and TrAA9A-treated BMCC. Cellulose loading was 2 mg/mL and total cellulase loading was 100 μg/mL with TlCel7A: EG: BG ratio of 6:3:1. The reactions in triplicate were conducted in 50 mM, pH 4.8, sodium acetate buffer at 150 rpm, 50°C for 72 h. [file 13068_2018_1023_MOESM11_ESM.jpg]
